# Gasdermins: a dual role in pyroptosis and tumor immunity

**DOI:** 10.3389/fimmu.2024.1322468

**Published:** 2024-01-18

**Authors:** Jiayi Yang, Jingting Jiang

**Affiliations:** ^1^Department of Tumor Biological Treatment, The Third Affiliated Hospital of Soochow University, Changzhou, Jiangsu, China; ^2^Jiangsu Engineering Research Center for Tumor Immunotherapy, The Third Affiliated Hospital of Soochow University, Changzhou, Jiangsu, China; ^3^Institute of Cell Therapy, The Third Affiliated Hospital of Soochow University, Changzhou, Jiangsu, China

**Keywords:** gasdermin, pyroptosis, caspase, granzyme, tumor immunity, immunotherapy

## Abstract

The gasdermin (GSDM) protein family plays a pivotal role in pyroptosis, a process critical to the body’s immune response, particularly in combatting bacterial infections, impeding tumor invasion, and contributing to the pathogenesis of various inflammatory diseases. These proteins are adept at activating inflammasome signaling pathways, recruiting immune effector cells, creating an inflammatory immune microenvironment, and initiating pyroptosis. This article serves as an introduction to the GSDM protein-mediated pyroptosis signaling pathways, providing an overview of GSDMs’ involvement in tumor immunity. Additionally, we explore the potential applications of GSDMs in both innovative and established antitumor strategies.

## 1 Introduction

The gasdermin (GSDM) protein family encompasses GSDMA/B/C/D, GSDME (DFNA5), and DFNB59 (pejvakin, PJVK) (1). These key molecules play a pivotal role in puncturing the cell membrane, releasing immune factors, and inducing cell death (1, 2). GSDM perforation is mediated by caspases and granzymes (GZMs), triggered through inflammasome signaling pathways, and holds critical significance in immune defense against pathogens and cancers (2). With the exception of DFNB59, all conserved proteins comprise an N-terminal punching domain and a C-terminal self-inhibition domain (3). Under normal conditions, these proteins cluster through domain interactions, inhibiting the perforating function of GSDM (3). Upon activation by pathogenic or damaging signals, caspases or GZMs cleave GSDM, separating it into N-terminal and C-terminal segments (4). These segments then oligomerize, forming pores in the cell membrane, resulting in the release of inflammatory molecules and cell pyroptosis (4, 5).

Pyroptosis, characterized by inflammation, is facilitated by GSDM proteins (6, 7). It manifests abruptly, eliciting a heightened inflammatory reaction compared to other programmed cell death mechanisms (8). In 2015, the segmentation of GSDMD into N-terminal and C-terminal domains by caspase-1 was discovered, revealing the pyroptosis process (9). The free N-terminal domain of GSDMD forms a channel in the cell membrane, causing an influx of water into the cell, altering osmotic balance, and leading to cellular expansion and membrane rupture (10, 11). Consequently, internal inflammation-inducing agents, such as interleukin-18(IL-18) and IL-1β, disperse, triggering an inflammatory reaction and forming an inflammatory microenvironment (4).

In recent years, immunotherapy has assumed a pivotal role in tumor treatment (12). Major modalities encompass oncolytic virus therapies, cancer vaccines, adoptive cell transfer, cytokine therapies, and immune checkpoint inhibitors (ICIs) (12). Studies demonstrate that combining programmed death-1/programmed death ligand 1 (PD-1/PD-L1) checkpoint blockade immunotherapies with the anti-angiogenic agent bevacizumab significantly enhances overall survival in non-small cell lung cancer (NSCLC) patients (13). Notably, this combination exhibits fewer adverse reactions compared to conventional chemotherapy drugs (13). Furthermore, in conjunction with tyrosine kinase inhibitors, ICIs have emerged as a pivotal aspect of renal cell carcinoma treatment (14). Despite remarkable progress, challenges persist, including limited applicability, unpredictable clinical effects, and innate or adaptive drug resistance issues, impeding broader clinical adoption of immunotherapy (15, 16). Resistance to innate immunotherapy may stem from a lack of intrinsic immune response to specific developing tumors, often observed in patients with systemic immunosuppression (16). Acquired immunotherapy resistance primarily reflects the tumor entering a therapeutic equilibrium state, closely tied to the intrinsic immune escape mechanisms of tumors (16). Pyroptosis, mediated by GSDM-related signaling pathways, not only induces tumor cell pyroptosis but also mitigates drug resistance and adverse reactions in treatment (7).

Research suggests that pyroptosis has dual implications for tumor immunity (17, 18). On the one hand, inducing pyroptosis in healthy cells releases inflammatory agents, creating an environment conducive to tumor growth and potentially prompting normal cells to undergo malignant transformation (19, 20). Conversely, an optimal level of pyroptosis ensures extracellular stability, enhances immune functionality, eliminates harm and pathogens, and safeguards the host (18). This review initiates with an exploration of the GSDM protein structure, followed by a detailed analysis of diverse activation mechanisms, associated regulatory components, and the engaged signaling pathways. Drawing insights from recent studies, we evaluate the dual functionality of the GSDM family concerning the tumor microenvironment, as well as the challenges and potential applications in tumor immunotherapy. Additionally, we underscore directions for future research. Notably, DFNB59, devoid of associations with pyroptosis induction, is not considered within the GSDM family under discussion.

## 2 Structure of GSDM

All proteins within the GSDM family, excluding DFNB59, feature two distinct domains: the C-terminal inhibitory domain (CT) and the N-terminal effector domain (NT) (21). The NT domain possesses cytotoxic properties (21). In the absence of cleavage initiation, the CT domain binds to the NT, thereby inhibiting its function (4, 22, 23). Upon enzymatic removal of the CT, the NT domain interacts with lipid molecules, forming pores in cellular membranes and facilitating the release of inflammatory proteins, including IL-1β, IL-18, IL-6, interferon γ (IFN-γ), etc. (24, 25). Notably, GSDMB stands out among its family members, demonstrating specific biological functions even in its full-length form, including a direct binding capability to phospholipids (26, 27). Cell viability is temporarily maintained through the recruitment of the endosomal sorting complexes required for transport (ESCRT) induced by calcium influx, removing GSDMD pores from the cell surface (17, 19, 28). Studies propose that inhibiting ESCRT-mediated cell membrane repair can enhance GSDM-triggered tumor pyroptosis (29). Other released contents encompass cellular alarms such as ATP and high mobility group box-1 protein (HMGB1), chemokines, and various cytokines, the specific types of which may vary depending on cell type and stimulus (23). These signals amplify inflammation in the tissue and recruit immune cells to respond to infection or danger (23). Furthermore, both GSDMD-NT and GSDME-NT can translocate to mitochondria, disrupting mitochondrial function, inducing reactive oxidative species (ROS) production, ultimately activating caspase-3, and enhancing pyroptosis (30).

## 3 Signaling pathways of GSDM-meditated pyroptosis

**Figure 1:**
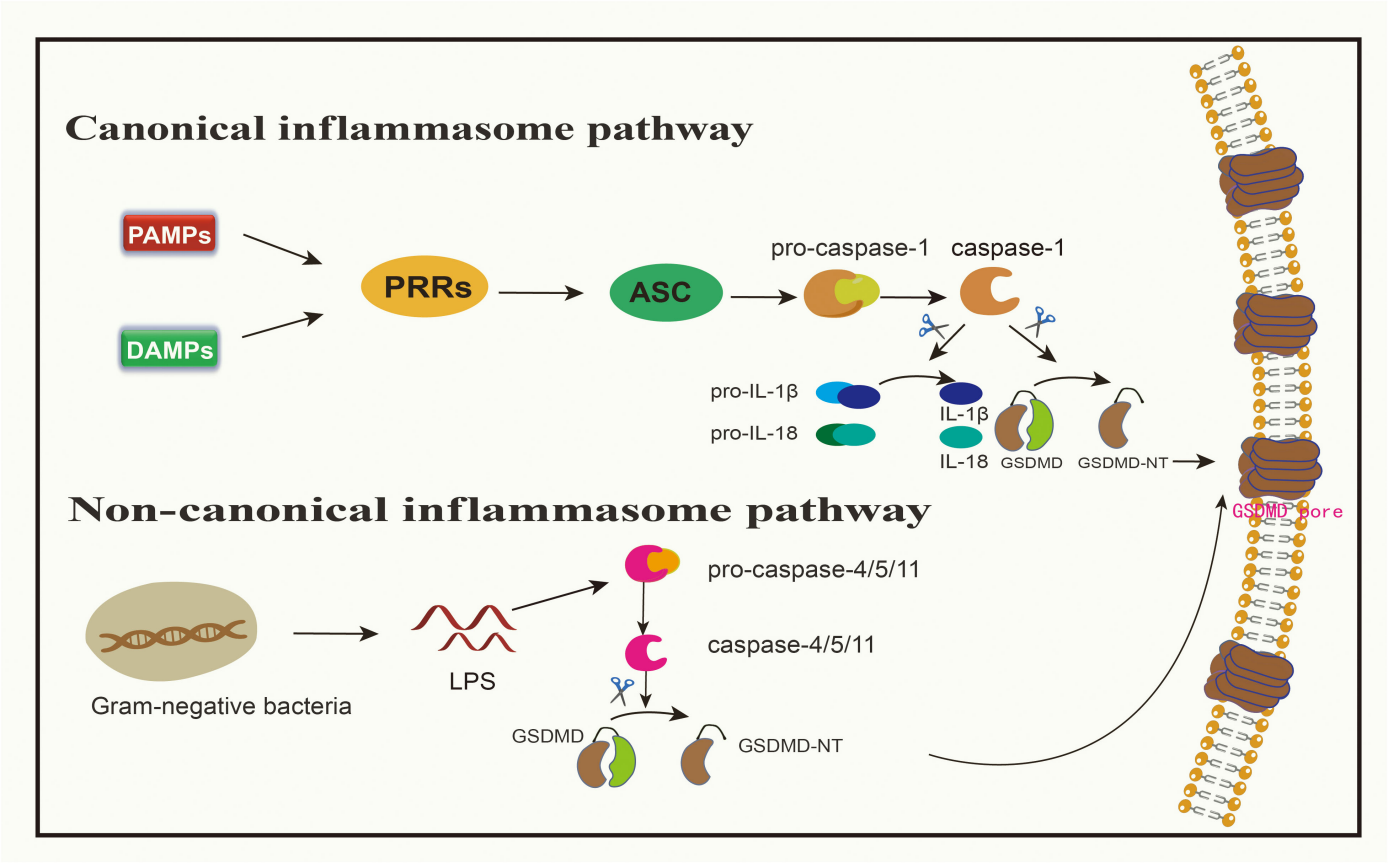
Canonical inflammasome pathway and non-canonical inflammasome pathway medidated by GSDMD. In canonical signaling pathway, when PAMPs or DAMPs bind to PRRs, ASC can be activated, resulting in the activation of pro-caspsase-1 into caspsase-1. Caspase-1 can cleave GSDMD to produce GSDMD-NT, and can also mature pro-IL-1β and pro-IL-18 into IL-1β and IL-18, allowing these inflammatory factors to spread from the channels formed by GSDMD-NT. In non-canonical signaling pathway, LPS in gram-negative bacteria can directly cleave pro-caspase-4/5/11, and activated caspase-4/5/11 cleave GSDMD to produce GSDMD-NT.

Pyroptosis follows two main pathways: the canonical and non-canonical inflammasome pathways, with GSDMD serving as the substrate for both (28, 31) (**Figure 1**). The caspase-1-dependent pathway is considered conventional, while activation involving human caspase-4/5 or rodent-specific caspase-11, initiated by bacterial lipopolysaccharide (LPS), is labeled unconventional (30). Additionally, other caspases-mediated and pyroptosis-associated signaling pathways exist. For instance, caspase-3 cleaves GSDME, and GSDMB undergoes segmentation by caspase-3/6/7 (3). The primary cleavage agents for GSDMA are yet to be identified. Furthermore, granzyme A and B, death-inducing proteases from natural killer (NK) cells and cytotoxic T lymphocytes (CTLs), cleave GSDMB and GSDME, respectively, activating pyroptosis (32–34). Granzymes exhibit dual functions: direct cleavage of GSDM proteins and stimulation of caspases to perform segmentation (35). Each GSDM protein has a distinct cleavage site, specifying unique initiating caspases or enzymes for each.

### 3.1 The canonical inflammasome pathway

Pattern recognition receptors (PRRs) discern pathogenic patterns, known as pathogen-associated molecular patterns (PAMPs) or non-pathogenic signals termed damage-associated molecular patterns (DAMPs), both capable of triggering pyroptosis (30). PAMPs encompass various biomolecules, including LPS on bacterial exteriors, flagellins, and both double- and single-stranded DNA and RNA in the cytoplasm or lysosomes introduced by viral incursions (36). DAMPs, on the other hand, primarily consist of bioactive entities released post-cellular damage or death, examples being ATP, the chromatin-linked protein HMGB1, and uric acid (37).

Toll-like receptors (TLRs), nucleotide-binding oligomerization domain-like receptors (NLRs), and absent in melanoma 2 (AIM2) all belong to the PRR category involved in pyroptosis (18, 20). When PRRs bind to either PAMPs or DAMPs, this engagement stimulates apoptosis-associated speck-like protein (ASC), which then forms oligomers (38). Subsequently, using its caspase activation and recruitment domain (CARD), it attaches to pro-caspase-1 (30, 38, 39). This complex, involving PRRs, ASC, and pro-caspase-1, is identified as the inflammasome (17, 38). This engagement leads to the conversion of pro-caspase-1 into its active form, caspase-1 (30). Notably, NOD-like receptor family pyrin domain 1(NLRP1) does not require ASC and can thus activate pro-caspase-1 directly without ASC oligomerization (40). Besides releasing and activating the N-terminal domain of GSDMD, caspase-1 also facilitates the maturation of pro-IL-1β and pro-IL-18 into IL-1β and IL-18, respectively, released through pyroptotic channels created by GSDMD-N (11, 39). Summarily, the pyroptosis instigated by inflammation is typified as a caspase-1-dependent inflammatory route, with GSDMD being the primary target of pro-inflammatory caspases (22). Studies targeting GSDMs have been developed, such as the antitumor effect in breast cancer via the caspase-1/GSDMD pathway (41). In research by Honglin Yan and colleagues, it is revealed that cisplatin enhances the activation of NLRP3 inflammasome by augmenting the expression of maternally expressed gene 3 (MEG3) (41). This then spurs GSDMD-driven pyroptosis via caspase-1 activation, resulting in the demise of triple-negative breast cancer cells (41).

### 3.2 The noncanonical inflammasome pathway

Bacterial LPS derived from gram-negative bacteria has the capacity to directly stimulate human caspase-4/5 or its murine counterpart, caspase-11 (42, 43). The CARD domain of pro-caspase 4/5/11 engages with the lipid fragment of LPS, inducing a notable conformational rearrangement (17). These conformational changes prompt the oligomerization of their CARD domains, facilitating the activation of caspase 4/5/11 (17). Upon activation, caspase-4/5/11 fragments GSDMD, yielding an N-terminal domain that breaches the cell’s outer barrier, thereby inducing pyroptosis (19, 43, 44). Simultaneously, this mechanism activates and releases IL-1β and IL-18, although their activation is driven by caspase-1 and not caspase-4/5/11 (45).

The non-canonical pathway intersects with the canonical pathway through NLRP3, activating pro-caspase-1 and cleaving pro-IL-1β/IL-18 to promote an inflammatory response (30). These events collectively trigger downstream effects, including cell membrane destruction and cytokine release through the GSDMD-N pore (46). Moreover, the attachment of the full-length GSDMB to the CARD domain of caspase-4 may induce the oligomerization of the caspase-4 protein (47, 48). Consequently, this boosts the fragmentation of GSDMD, igniting an alternative pyroptosis pathway (47, 48). Throughout this alternative pyroptotic process, any influence of GSDMB on caspase-4 can be halted by a built-in counteracting mechanism, ensuring that sustained pyroptosis doesn’t inflict excessive damage (49). The activation of the inflammasome through both canonical and non-canonical signaling pathways empowers the host to deploy various defense mechanisms depending on the type of pathogen (17).

### 3.3 Other signaling pathways

**Figure 2:**
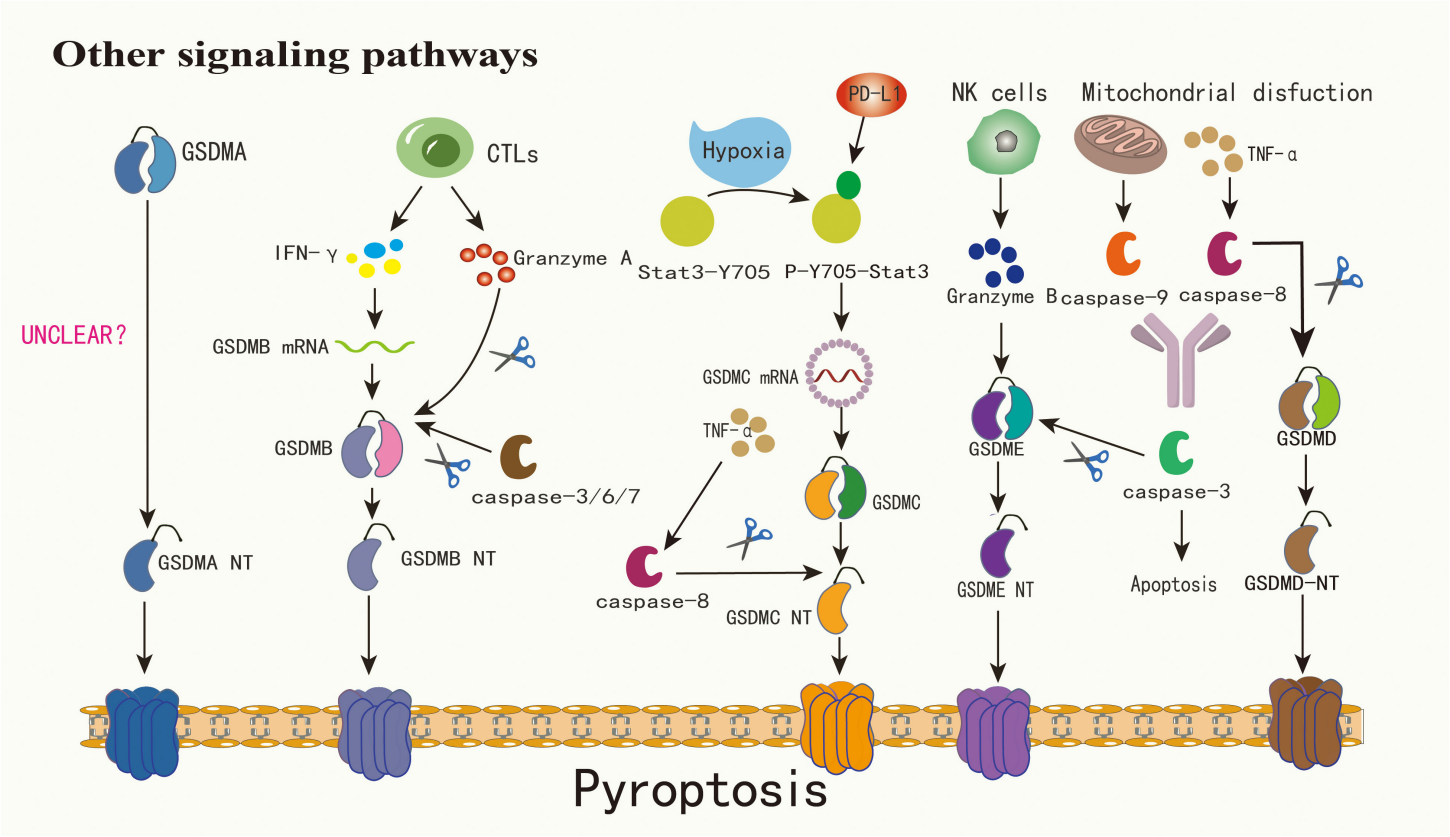
The mechanism by which GSDMA is cleaved into GSDMA-NT warrants further investigation. Cytotoxic lymphocytes have the capability to secrete granzyme A and IFN-γ, with the latter notably enhancing GSDMB expression and subsequently facilitating its cleavage by granzyme A. Concurrently, caspase-3/6/7 can cleave GSDMB, triggering pyroptosis. Under hypoxic conditions, Stat3-Y705 undergoes phosphorylation, transforming into p-Y705-Stat3. This form of Stat3 interacts with PD-L1 and fosters the transcription of GSDMC within the nucleus, thereby amplifying its expression. Activation of caspase-8, induced by TNF-α, leads to the cleavage of GSDMC into GSDMC-NT. Similarly, GSDMD is subject to cleavage by caspase-8. Additionally, granzyme B, secreted by NK cells, can directly cleave GSDME into GSDME-NT. Mitochondrial dysfunction can precipitate the release of caspase-9, which along with caspase-8, can activate caspase-3, culminating in apoptosis. In contexts characterized by elevated GSDME expression, TNF-α presence, or exposure to chemotherapeutic agents, caspase-3 is capable of cleaving GSDME, thereby inducing pyroptosis

Other inflammatory protein-mediated pyroptotic signaling pathways also play a significant role in immune defense (**Figure 2**). Caspase-8, derived from tumor necrosis factor α (TNF-α) secreted by immune cells, can trigger GSDMD-dependent pyroptosis (50). NETosis, a regulated form of neutrophil death activated by microbial infections and other danger signals, involves the release of large networks of genomic DNA, histones, and antimicrobial proteins, known as neutrophil extracellular traps (NETs) (3, 25, 28). Serine proteases, such as neutrophil elastase (ELANE), released from granules, cleave and activate GSDMD, initiating NETs (3, 23).

Under specific scenarios, such as exposure to TNF-α, certain cancer treatment medications, or elevated levels of GSDME, caspase-3 can initiate GSDME-associated pyroptosis (22, 24). Although GSDME is found only in a limited subset of cancer cells, it plays a pivotal role in modulating the immune environment within tumors, promoting a robust T-cell-driven counter-cancer immune response (51, 52). Beyond the caspase-driven method, tumors with heightened GSDME, when paired with NK cells, demonstrate that granzyme B(GZMB) from these NK cells effectively segments GSDME, triggering the GSDME-related pyroptosis pathway (22, 37). Intriguingly, GZMB also stimulates caspase-3 to segment GSDME, establishing a reinforcing antitumor cycle and contributing to tumor inhibition (39). In addition to GZMB, another serine protease derived from cytotoxic lymphocytes, granzyme A(GZMA), can segment GSDMB and induce pyroptosis in tumor cells (2, 32, 53). Due to the lack of a distinct junction region, GSDMB also becomes a target for caspase-3/6/7 segmentation (18). The exact function of GSDMB in pyroptosis remains a matter of debate. GSDMA-NT, GSDMD-NT, and GSDME-NT display comparable pore-creation capabilities. Yet, the activation process for GSDMA remains unidentified (2). GSDMC is prominently expressed in advanced melanoma and undergoes cleavage by caspase-8 (43, 54). Agents like PD-L1, TNF-α sourced from macrophages, certain medications, and cancer treatments can induce breast cancer cell pyroptosis via the caspase-8/GSDMC route (19). The complex signaling pathways of pyroptosis provide a new direction for regulating tumor immunity: by targeting inflammatory factors upstream and downstream of these pathways or activators, such as GZMs, we can modify the tumor microenvironment caused by pyroptosis.

## 4 The expression of GSDM and clinical significance in tumor immunity

The GSDM proteins are predominantly expressed in the epithelium of the esophagus, stomach, colon, bladder, vagina, and prostate (29, 55). Their presence in different tumors varies significantly, contributing to distinct roles within these tumors (56, 57) (**Table 1**).

**Table 1:** The signaling pathways of GSDM proteins and their effect on different tumors.

Gasdermin genes		Structure	Signaling pathways	Tumor	Relative expression	Associated functions	References
Human	Mouse						
GSDMA	GsdmA1 GsdmA2 GsdmA3	the C-terminal inhibitory domain and the N-terminal effector domain	Activation pathway remains unknown Upregulated by TGF-β	Esophageal cancer	Decrease	Promote the growth of tumor cells	(58)
				Gastric cancer	Decrease	Promote the growth of tumor cells	(23)
				Lung cancer	Increase	Unclear	(19)
GSDMB	None	the C-terminal inhibitory domain and the N-terminal effector domain	Cleaved by GZMA, caspase-3/6/7 Upregulated by IFN-α, IFN-β, IFN-γ, TNF-α	Breast cancer	Increase	Accelerate tumor progression	(59)
				Gastric cancer	Increase	Facilitate the proliferation of tumor cells	(60)
				Colorectal cancer	Increase	Inhibit the growth of tumor cells and damage the epithelial barrier function	(26)
				Cervical cancer	Increase	Accelerate metastasis	(61)
				Clear cell renal cell carcinoma	Increase	Indicate high TNM stage and poor prognosis	(62)
GSDMC	GsdmC1 GsdmC2 GsdmC3 GsdmC4	the C-terminal inhibitory domain and the N-terminal effector domain	Cleaved by caspase-8 Downregulated by TFG-β Upregulated by STAT3-PDL1	Lung cancer	Increase	Indicate poor prognosis	(63)
				Colorectal cancer	Increase	Accelerate the proliferation of tumor cells and promote carcinogenesis	(64)
				Pancreatic cancer	Increase	Indicate poor overall survival	(65)
				Melanoma	Increase	Indicate tumor progression and poor overall survival	(27)
				Clear cell renal cell carcinoma	Increase	Indicate poor overall survival	(54)
				Gastric cancer	Increase	Inhibit the growth of tumor cells	(19)
GSDMD	GsdmD	the C-terminal inhibitory domain and the N-terminal effector domain	Cleaved by caspase-1/4/5/11/8, neutrophil elastase Downregulated by promoter DNA methylation	Gastric cancer	Decrease	Inhibit the proliferation of tumor cells	(66)
				Endometrial carcinoma	Increase	Exert anti-tumor immune effect	(67)
				Colorectal cancer	Decrease	Inhibit the growth of tumor cells	(68)
				Hepatocellular carcinoma	Increase	Accelerate carcinogenesis	(69)
				Lung adenocarcinoma	Increase	Promote the growth of tumor cells and accelerate metastasis	(70)
				Osteosarcoma	Increase	Cause drug resistance and poor prognosis	(71)
				Glioma	Increase	Promote the invasion and proliferation of glioma stem cells	(72)
GSDME /DFNA5	GsdmE	the C-terminal inhibitory domain and the N-terminal effector domain	Cleaved by GZMB, caspase-3 Upregulated by p53 Downregulated by promoter DNA methylation	Colorectal cancer	Decrease	Inhibit colony formation and cell proliferation	(73)
				Breast cancer	Decrease	Improve the prognosis and reduce the mortality	(74)
				Gastric cancer	Decrease	Inhibit colony formation and cell proliferation	(73)
				Melanoma	Decrease	Enhance the killing effect of cytotoxic lymphocytes	(75)

### 4.1 Effect of GSDM-mediated pyroptosis on tumor microenvironment

GSDM-mediated pyroptosis serves as a double-edged sword in the tumor microenvironment (19). In general, moderate pyroptosis can inhibit tumor growth, but excessive levels may have the opposite effect (19). Striking a balance between the cancer-promoting and cancer-inhibiting effects of pyroptosis poses a challenging problem for future research. The reshaping of the tumor microenvironment through pyroptosis has the potential to mitigate drug resistance among tumor cells (34, 76).

Inflammatory factors released during pyroptosis, such as IL-1β and IL-18, exhibit dual roles in fostering and targeting tumors (11, 46). Investigations reveal that tumors display elevated expression of IL-1β compared to regular tissues (37). This heightened level can enhance tumor cell proliferation, invasion, and metastasis by promoting the expression of vascular endothelial growth factor A (VEGFA) and angiogenesis (37, 46). However, IL-1β can also induce dendritic cell (DC) maturation and promote the differentiation of monocytes into DCs and inflammatory macrophages (19). Mature DCs can present tumor antigens to CTLs and eliminate tumor cells (19). In the inflammatory microenvironment formed by pyroptosis, over-activated DCs can release a substantial amount of IL-1β, creating positive antitumor immune feedback (46). Tumor-derived macrophages (TAMs) secrete transforming growth factor β (TGF-β), IL-10, and arginase I, inhibiting the cytotoxic function of T cells, NK T cells, and NK cells (77). TAMs also express high levels of chemokines, such as CCL5 and CCL20, recruiting natural regulatory T (nTreg) cells to suppress antitumor immunity (77). Tregs exhibit immunosuppressive activity through high expression of cytotoxic T lymphocyte-associated protein (CTLA)-4, secretion of inhibitory cytokines(IL-10, TGF-β), and degradation of ATP (77). Furthermore, IL-1β can drive antigen-specific cytotoxic CD8^+^T cell responses, increase the number of Th1 cells, and inhibit the differentiation of immunosuppressive Treg cells (19). ATP released by pyroptotic tumor cells can activate the NLRP3 inflammasome of DCs, activate CD8^+^ T cells, and enhance IL-1β-dependent antitumor immunity (46). Similarly, while the overexpression of IL-18 in tumors may enhance malignant behavior and typically indicate an unfavorable prognosis, it can also modulate the immune system, hindering tumor progression by recruiting NK cells, T cells, and monocytes (78, 79).

Pyroptotic cells also release HMGB1 (23). On the one hand, HMGB1 can promote the maturation of DCs, subsequently activating CTLs to eliminate tumor cells (79, 80). On the other hand, HMGB1 interacts with TLR2 and TLR4 receptors on the surface of various immune cells, activating the transcription factors NF-κB and activator protein-1(AP-1), thereby promoting the secretion of inflammatory cytokines such as IL-6 and TNF-α (81). Additionally, it drives the accumulation of myeloid-derived suppressor cells (MDSCs) by inducing autophagy, maintaining the activity of MDSCs, and ultimately inhibiting antitumor immunity (82, 83). The chemotherapy drug gemcitabine can induce MDSC pyroptosis, releasing IL-1β and reducing anticancer immunity by inducing CD4^+^T cells to secrete IL-17 (77). Further studies on the role and regulatory mechanisms of immunosuppressive cells are needed to modulate the tumor immune microenvironment effectively.

The expression of GSDME enhances the absorptive abilities of TAMs and amplifies the count and effectiveness of tumor-infiltrating NK cells and CD8^+^ T cells, along with the expression of key molecules (GZMB, IFN-γ, and TNF-α) in tumor-infiltrating lymphocytes (TILs), potentially bolstering antitumor adaptive defenses (84, 85). In certain tumors that evade immune responses by preventing absorption, GSDME offers a potential solution to this immune escape tactic (86). Melanomas, characterized as “cold” tumors due to their poor response to ICIs, can be transformed into “hot” tumors through the induction of GSDME-related pyroptosis, especially in GSDME-expressing BRAF^V600E/K^ mutant melanoma cells (87). In co-administration of B-Raf proto-oncogene (BRAF) and mitogen-activated protein kinase (MEK) inhibitors amplifies GSDME-related pyroptosis, leading to the release of HMGB1 (23, 87, 88). This escalation results in increased immune cell penetration into the tumor and subsequent tumor shrinkage (87). This suggests that triggering pyroptosis can potentially convert “cold” tumors into “hot” tumors, thereby altering the tumor immune microenvironment and promoting the antitumor effects of immune cells.

### 4.2 The effect of GSDME on tumor

GSDME, alternatively known as DFNA5, is situated on the 7p15.3 segment of the human chromosome (37). Its presence is predominantly observed in the cochlea, thymus, kidneys, brain, lungs, and digestive tract (36, 89). The expression of GSDME can be triggered by p53 via the p53-binding site in the intron 1 of GSDME, suggesting that it is a transcriptional target for the p53 family (52, 90). Methylation of the promoter DNA can lead to the epigenetic deactivation of GSDME in various cancer cell lines and primary tumors, and this methylation process of GSDME has been proposed as a valuable indicator for detecting cancers (64, 91).

GSDME has demonstrated inhibitory effects on cell proliferation and tumor progression in gastric cancer (GC), melanoma, colorectal cancer (CRC), and breast cancer, where its expression is upregulated, indicating its potential role as a tumor-suppressing agent (25, 34, 92). In the context of breast cancer, GSDME pyroptosis is primarily mediated by caspase-3 through the ROS/JNK signaling pathway (93). The methylation rate of the GSDME promoter in lobular carcinomas is notably higher than in ductal carcinomas (74, 85). Furthermore, this promoter methylation shows a clear association with tumor progression, peaking in stage III, while stages I and II exhibit equivalent rates of methylation (74). The methylation value of the GSDME promoter is also markedly elevated in PR^+^ compared to PR^-^ breast cancer (94).

Intrinsic levels of GSDME significantly impact the outcome of chemotherapy treatments (52, 95). The presence of GSDME in most tumor cells is rare (73). Decitabine has the ability to reverse GSDME silencing and sensitize tumor cells to chemotherapy drugs (51, 74, 91). Studies on the lung cancer cell line A549 have revealed that both cisplatin and paclitaxel induce pyroptosis via the caspase-3/GSDME signaling pathway, with cisplatin exhibiting a more pronounced effect than paclitaxel (64, 73, 90). In melanomas, the administration of doxorubicin can trigger caspase-3-driven pyroptosis via GSDME, and the antitumor efficacy is enhanced when the elongation factor-2 kinase (eEF-2K) is inhibited (75, 93). Additionally, research has identified that lobaplatin can initiate pyroptosis in CRC via GSDME, enhancing ROS levels and activating the c-jun N-terminal kinase (JNK), leading to caspase-3/9 activation through the ROS/JNK/BAX mitochondrial apoptosis pathway (75). Similarly, lobaplatin induces pyroptosis in cervical cancer cells (64). These insights open avenues for potential strategies using GSDM proteins in conjunction with chemotherapeutic agents to enhance antitumor immune responses.

The side effects of chemotherapy are also associated with GSDME-induced pyroptosis (91, 96, 97). Studies have shown that depletion of GSDME can reduce lung damage caused by agents like cisplatin or bleomycin in rodents (52). Additionally, research by Xiujin Shen and colleagues has revealed that cisplatin- or doxorubicin-induced GSDME-dependent pyroptosis leads to varying extents of renal impairment (96). The expression of GSDME is also linked to chemotherapy resistance (98, 99). In lung cancer, the absence of GSDME enhances resistance to treatment, while its overexpression augments drug susceptibility (52). Furthermore, melanoma cells exhibit increased resistance to etoposide with reduced GSDME expression (99). These findings underscore the importance of evaluating GSDME’s influence on chemotherapeutic agents to optimize its benefits in cancer therapy. Considering the adverse effects of high chemotherapy dosages on the human system, increasing GSDME expression may enhance the antitumor efficacy of these agents, potentially allowing for dosage reductions.

In addition to traditional chemotherapy drugs, targeted medications can exploit the GSDME-associated signaling pathway to exert therapeutic effects. For instance, harnessing microRNA to reactivate GSDME expression, in combination with cetuximab, has demonstrated promising results in initiating pyroptosis in malignant cells and reducing tumor size in mouse models of aggressive triple-negative breast cancer (34, 100). Furthermore, the use of polo-like kinase 1(PLK1) inhibitors alongside cisplatin in the treatment of esophageal squamous cell carcinoma has been shown to enhance the therapeutic response (34, 74). This enhancement is attributed to their role in promoting GSDME-related pyroptosis, thereby augmenting the effectiveness of chemotherapy (74, 75).

In summary, the application of GSDME represents an innovative frontier in tumor immunotherapy by inducing pyroptosis in cancer cells, exposing tumor antigens to the immune system, and thereby enhancing the antitumor immune response (94). This approach not only directly targets tumor cells but also modulates the tumor microenvironment to create a more conducive setting for an effective immune attack (94). It is crucial to explore the pyroptosis-related signaling pathways triggered by chemotherapy or targeted drugs through GSDME and to target the inflammatory factors via these pathways to fully exploit antitumor effects. Ensuring the safety of this combination therapy requires our in-depth consideration. The prospect of integrating GSDME into immunotherapeutic strategies offers a promising avenue for the development of more potent and specific cancer treatments, potentially improving outcomes for patients with various types of cancers.

### 4.3 The effect of GSDMB on tumor

The GSDMB gene is located on human chromosome 17q21.1, but it is notably absent in mice (47). This absence in mouse models has limited *in vivo* testing for GSDMB (47). GSDMB is predominantly found in the lungs, esophagus, digestive system, and immune cells (23, 27). Its expression can be enhanced by IFN-α, β, γ and TNF-α (32). Elevated levels of GSDMB often correlate with invasive tendencies in various cancers, including GC, breast cancer, cervical cancer, and CRC (101, 102). However, it’s essential to note that GSDMB can also exert antitumor effects (32). To fully harness the therapeutic potential of GSDMB, determining the optimal way to modulate its expression for the intended effect is crucial.

The expression of GSDMB in the intestinal mucosal epithelium may provide broad-spectrum defense against inflammatory bowel disease (IBD), CRC, and intestinal infections (26, 103, 104). Studies suggest that GSDMB accelerates epithelial tissue recovery primarily by enhancing the phosphorylation of focal adhesion kinase (FAK), a critical regulator in focal adhesion transformation (26, 105). The full-length form of GSDMB within intestinal epithelial cells (IECs) can minimize local adhesion complexes, weaken cell binding to the extracellular matrix (ECM), amplify IEC proliferation and mobility, and is beneficial for inflammation repair (105–107). The protective effect of GSDMB on the intestinal epithelium is further evidenced by its ability to mark cancerous or aberrant epithelial cells for pyroptotic elimination, ensuring comprehensive protection and maintaining intestinal regularity (26, 89). Conversely, an increase in GSDMB-dependent pyroptosis within the intestinal epithelium may compromise its barrier function, allowing bacterial movement into the submucosal layer and potentially amplifying chronic inflammation due to unchecked immune reactions (103, 108). In summary, monitoring GSDMB protein levels in the intestinal mucosa may serve as a valuable indicator for tracking the progression of IBD and CRC (103).

In contrast to its role in IBD and CRC, heightened GSDMB levels are intricately linked to the aggressive characteristics of breast cancer, specifically its invasive nature, progression, and metastatic potential (59, 100, 101). This positions GSDMB as a promising candidate for breast cancer detection and prognostic assessment (59). Research demonstrates that intracellular administration of GSDMB-specific antibodies can significantly and selectively hinder the metastasis, migration, and therapeutic resistance associated with human epidermal growth factor receptor 2 (HER2)-positive breast cancer (109). The N-terminal domain of GSDMB collaborates with pivotal components of protective autophagy, namely microtubule-associated protein light chain 3B (LC3B) and Rab7 (a small GTPase crucial for autophagosome maturation) (109, 110). This interaction fosters Rab7 activation, leading to diminished efficacy of targeted therapy in HER2-positive breast cancer cells (109, 110). Importantly, targeted inhibitors such as chloroquine (CQ) can suppress autophagy, restoring cancer cell sensitivity to therapeutic agents and amplifying cancer cell death (111). Therefore, combining anti-HER2 agents like trastuzumab with chloroquine may enhance therapeutic outcomes in cancers exhibiting both GSDMB and HER2 positivity (111). Altogether, this mechanistic insight offers groundbreaking avenues for enhancing immunotherapeutic strategies and prognosis monitoring tailored for HER2-positive breast cancer.

The expression of GSDMB experiences a marked upsurge in GC, implicating the potential role of GSDMB in modulating cancer cell proliferation (60, 112, 113). Notably, Komiyama et al. have identified an Alu element that augments GSDMB expression specifically within GC cells (114). In parallel, Saeki et al. have deciphered that an LTR-derived promoter also facilitates elevated GSDMB expression in GC cells (60). Synthesizing the findings from both studies, it’s conceivable that evaluating the expression of GSDMB in tandem with the presence of the Alu element or the LTR-derived promoter may serve as valuable markers for monitoring GC onset and trajectory (60, 114).

Persistent infections with carcinogenic strains of high-risk human papillomavirus are the primary culprits behind cervical cancer, a major cause of mortality in females (61). Elevated GSDMB levels are associated with the onset and progression of cervical squamous cell tumors, potentially enhancing their proliferation and hastening the spread of early-stage cancer cells (61). In a parallel context, both mRNA and protein levels linked to GSDMB increase in clear cell renal cell tumors (ccRCC) and show a strong correlation with elevated TNM classifications and a poor prognosis (62, 115, 116). Therefore, GSDMB could serve as a potential indicator for assessing adverse health trajectories in ccRCC patients, potentially playing a pivotal role in the body’s immune response (62, 116).

Generally, GSDMB exhibits a dual effect of promoting and inhibiting tumor progression. The specific effect should be identified based on its expression and mechanism in different organs and tissues (11). Additionally, the application of GSDMB in immunotherapy could synergize with existing therapies, such as ICIs, enhancing their efficacy (117). However, translating this potential into clinical practice requires careful investigation and understanding of the interaction of GSDMB-induced pyroptosis with various types of immune cells to ensure specificity for tumor cells and to avoid off-target effects.

### 4.4 The effect of GSDMD on tumor

GSDMD, located on human chromosome 8q24.3, is ubiquitously present across diverse cell types, including gastrointestinal epithelial cells, placental cells, immune cells (notably macrophages and DCs), and cancerous cells (36, 89, 118). The regulatory function of interferon regulatory factor 2 (IRF2) enhances GSDMD transcription, a crucial step for initiating pyroptosis (3, 39). Moreover, insights from the Cancer Genome Atlas highlight a relationship between GSDMD expression and CD8^+^ T cell indicators in primary tumors of several cancers (27). In activated human CD8^+^ T cells, GSDMD cleavage surges, while depletion of GSDMD diminishes the combative abilities of CD8^+^T cells (27, 58).

Primarily, GSDMD functions as a tumor-inhibiting agent (29). The downregulation of GSDMD expression in gastric cancer cells promotes tumor progression, possibly through the activation of the PI3K/PKB signaling pathway and the expedited transition of S/G2 cells (48, 66, 118). In cases of endometrial carcinoma, tumor cells display elevated expression levels of GSDMD compared to their healthier counterparts (67). This overexpression is associated with antitumor immune responses and a more favorable prognosis (67). When endometrial or ovarian cancer cells are exposed to the reversible acetylcholine transferase inhibitor α-NETA, there is an induction of pyroptosis through the GSDMD/caspase-4 mechanism, leading to suppressed tumor growth in mouse models (64, 90). Additionally, a reduced presence of GSDMD predicts an unfavorable outcome for CRC patients (68, 119). Hence, GSDMD emerges as a pivotal indicator of disease progression and a promising focal point for CRC treatments (68, 119).

GSDMD has also been identified as having cancer-promoting properties (30). Recent research has revealed that aged hepatic stellate cells persist in displaying a senescence-associated secretory phenotype (SASP), releasing proteins like IL-33, which counteract antitumor immunity (69). Hepatic lipid metabolism triggers the cleavage of GSDMD, forming cell membrane pores and facilitating the release of IL-33 (69). This process intensifies the onset of obesity-related hepatocellular carcinoma (HCC) (69). From this, it can be inferred that curbing IL-33 and GSDMD may impede the progression of obesity-driven HCC (69, 120, 121). Moreover, high expression of GSDMD in lung adenocarcinoma (LUAD) is associated with larger tumors, advanced lymph node tumor stage, and poor prospects (70, 122). However, the expression of GSDMD doesn’t show any linkage with survival rates in lung squamous cell carcinoma (LUSC), suggesting the potential of GSDMD as a standalone prognostic indicator for LUAD (70). Stifling GSDMD scales down the progression of tumor cells by hampering the EGFR/Akt signaling pathway (122). Interestingly, simvastatin has the potential to activate the NLRP3/caspase-1 cascade, leading to pyroptosis in LUAD cells (123). This finding implies that simvastatin, known for treating high cholesterol, may be repurposed as a treatment targeting GSDMD in lung cancer (123). The expression of GSDMD is significantly upregulated in osteosarcoma and is associated with drug resistance and poor prognosis in osteosarcoma patients, providing new insights into the diagnosis and prognosis prediction of osteosarcoma (71, 124). Researchers have produced dichloroacetate polymer micelles (OPDEA-PDCA) that can induce mitochondrial oxidative stress by inhibiting pyruvate dehydrogenase kinase 1 (PDHK1), leading to GSDMD-mediated pyroptosis of osteosarcoma cells and enhancing osteosarcoma immunotherapy (125). In addition, this polymer can stimulate the secretion of PD-L1, indicating that the combination of this polymer with a PD-L1 monoclonal antibody can markedly inhibit the proliferation of osteosarcoma and induce the activation of T cells (125). As the most common malignant tumor in the central nervous system, glioma has a very poor prognosis (126). Pyroptosis induced by GSDMD and NLRC4 in high-grade gliomas can promote the progression of gliomas and enhance the sensitivity of these tumors to immune checkpoint therapy by recruiting immune cells (127). The inflammatory immune microenvironment induced by pyroptosis can promote the invasion and proliferation of glioma stem cells and is also related to the infiltration of M2 pheno-type bone marrow-derived macrophages (BMDMs) (72, 128). In the future, it is necessary to construct a GSDMD-deficient mouse model to probe the effects of pyroptosis as a drug target on gliogenesis and the tumor immune microenvironment (72, 127).

GSDMD is involved in various pyroptosis signaling pathways, highlighting its intricate role in mediating the pyroptosis of tumor cells. This complexity underscores the challenge of precisely delineating the mechanisms through which GSDMD induces pyroptotic cell death within the tumor microenvironment. Consequently, substantial efforts have been directed toward developing pharmacological agents that target pathways associated with GSDMD (3). Specifically, there is an exploration into antagonists designed to inhibit GSDMD activity, aiming to effectively modulate tumor immune responses (3). Additionally, key components of the pyroptosis pathway, such as NLRP3 and caspase 1/4/5/11, are also focal points for drug development (30). Targeting these molecules provides another strategic avenue to manipulate the pyroptotic pathway, potentially offering new therapeutic options for cancer treatment.

### 4.5 The effect of GSDMC on tumor

The GSDMC gene is located on human chromosome 8q24.21 and is predominantly expressed in keratinocytes and the digestive system (58, 89). Its role in tumor immunity varies depending on the organ and tissue context (63). Initially, abnormal up-regulation of GSDMC is identified in metastatic melanoma, earning it the label of melanoma-derived leucine zipper extra-nuclear factor (MLZE) and making it a molecular beacon for tracking melanoma evolution (27, 65).

In CRC, the transforming growth factor-β receptor II (TGF-βR2) often displays deactivating alterations (57). This results in a surge in GSDMC, promoting the growth of tumor cells and fostering tumorigenesis, positioning GSDMC as a potential therapeutic target for TGFβR2 mutant CRC (57). Moreover, GSDMC undergoes hypomethylation in LUAD cells, and elevated levels usually hint at a grim prognosis for LUAD patients (63). In pancreatic cancer, GSDMC’s presence is nearly double compared to healthy tissues, correlating with reduced patient longevity (65, 129). Research led by Yun-Qian Cui indicates pronounced expression of GSDMC in ccRCC, linking it to adverse survival rates (54). This makes it a suitable diagnostic, prognostic, and therapeutic touchpoint for ccRCC (54). However, certain studies have pinpointed the ability of GSDMC to suppress the progression of esophageal squamous cell carcinoma and GC, underscoring its potential antitumor capabilities (19). The intricate dynamics of GSDMC in cancer development warrant deeper exploration.

In oxygen-deprived environments, Stat3-Y705 undergoes phosphorylation, resulting in p-Y705-Stat3 (21, 37). This molecule can associate with PD-L1, converting TNF-α-induced apoptosis into pyroptosis, primarily within the cell nucleus (21, 37). Mien-Chie Hung and colleagues have elucidated that the nPD-L1/p-Y705-Stat3 complex targets the Stat3 binding domain on the GSDMC promoter, initiating GSDMC mRNA transcription and increasing GSDMC levels (21). In tumor cells, TNF-α triggers caspase-8, leading to GSDMC cleavage at the D365 amino acid (50, 55). The N-terminal domain of GSDMC then inserts itself into the cell membrane, resulting in cellular disruption (55). Certain antibiotic chemotherapy agents have been observed to elevate the levels of nPD-L1 and GSDMC while inducing caspase-8, leading to pyroptosis within tumor cells (46).

### 4.6 The effect of GSDMA on tumor

The human GSDMA gene is located on chromosome 17q21.1, while in mice, the genes Gsdma1, Gsdma2, and Gsdma3 are clustered on chromosome 11D (64). GSDMA expression is predominantly observed in the epithelial layer of the skin and the lungs (19). Its unique regulation is governed by the transcription factor LIM domain 1, triggered by the action of TGF-β (27, 112). Notably, GSDMA is silenced in esophageal cancer and GC cells, primarily attributed to epigenetic modifications, particularly methylation, in mucin-secreting pit cells of the gastric epithelium (130).

In an innovative cancer-combating approach, Wang and colleagues have utilized a combination of the cancer imaging probe phenylalanine trifluoroborate (Phe-BF3) with gold nanoparticles (NPs) (131). This combination effectively delivers the mouse version of GSDMA, specifically Gsdma3, to various human and mouse cancer cells, including HeLa (cervical), EMT6 (mammary), and 4T1 (mammary) cells (131). The study results highlight the potential of Gsdma3 in inducing pyroptosis of tumor cells and promoting pyroptosis-related immunotherapy, with this effect appearing contingent upon the activity of the immune system (131). This underscores the complex interplay between pyroptotic pathways and the host immune response, strategically manipulable for therapeutic benefits in cancer management.

## 5 Conclusions and perspectives

The GSDM proteins play a pivotal role in orchestrating pyroptosis, a process integral to tumor immunity. These proteins enhance the activity and permeation of immune cells by releasing cytokines and immune-responsive agents upon cellular disintegration. Pyroptosis operates through a dual-role mechanism that can both foster and restrain tumor proliferation. On the one hand, the inflammatory agents liberated during pyroptosis can invigorate the immune response, leading to the death of cancerous cells. On the other hand, pyroptosis can establish an inflammation-rich milieu that augments cancer cell proliferation. Delving deeper into how pyroptosis functions across various cancer cells, along with the proteins that precede and follow the GSDM signaling cascade, can illuminate novel therapeutic avenues for these malignancies.

Presently, researchers have formulated numerous drugs that influence pyroptotic signaling pathways, with a particular focus on agonists or antagonists targeting GSDMD. These medications can impact the release of inflammatory factors during the pyroptosis process, offering novel avenues for treating both inflammatory diseases and malignant tumors. DSF (Disulfiram) is an FDA-approved drug for the treatment of chronic alcoholism that blocks pore formation by covalently modifying Cys191 in GSDMD, inhibiting the pyroptosis process and cytokine release, but does not affect caspase activation or GSDMD cleavage. Different from DSF, DMF (Dimethyl fumarate) can prevent GSDMD from interacting with caspases, limit GSDMD oligomerization and cleavage. Although pyroptosis holds promise as a potential avenue for cancer treatment, its application in tumor immunotherapy is still in its early stages and encounters various challenges. These challenges include issues of selectivity, resistance, inflammatory responses, and variability in effectiveness across different cancer types. As a pan-caspase inhibitor, Z-VAD-FMK was originally developed for irreversibly binding and specific inhibition of inflammatory caspase 1/4/5/11. However, it has also been found to inhibit caspases 3/7/8, thus inhibiting pyroptosis and apoptosis. Future research efforts should concentrate on investigating ways to precisely manipulate pyroptosis-related inflammatory factors and caspases, such as NLRs and caspase-1, to avoid off-target effects. Currently, a combination of nanoparticles, pyroptotic inducers, and tumor cell markers has been developed to selectively induce pyroptosis of malignant cells *in vivo*. Studies have shown that intravenous NPs, acting in concert with immune checkpoint blockade, can sensitizing tumors to anti-PD-1 therapy, suggesting that pyroptosis can be exploited to trigger or enhance anti-tumor immunity. Immunotherapy grapples with challenges such as innate and acquired drug resistance. How to harness the modifying effect of pyroptosis on the tumor immune microenvironment to enhance the response of tumor cells to drugs is a topic worthy of profound consideration. Moreover, achieving a balance in regulating pyroptosis, striking the right equilibrium for the desired antitumor effect while ensuring the safety of this immunotherapy approach and mitigating adverse side effects of the inflammatory cascade reaction, such as cytokine release syndrome (CRS), has the potential to elevate pyroptosis in the treatment of tumors to a new level.

Therapies centered on GSDM, particularly those targeting the GSDME protein within the GSDM family, represent a significant breakthrough in cancer treatment. GSDME primarily functions as a tumor suppressor, effectively inhibiting the invasion and proliferation of tumor cells. However, its expression often diminishes in tumors, primarily due to GSDME mRNA methylation—a phenomenon that can serve as a predictive marker for cancer. GSDM proteins, including GSDME, can serve as reliable biomarkers associated with pyroptosis induction in tumors, aiding in patient selection for treatments and monitoring treatment responses. During chemotherapy, drugs like gemcitabine can revive suppressed GSDME, promoting pyroptosis and potentially reducing the side effects of chemotherapy drugs while offering protection against drug resistance. It’s crucial to note, however, that pyroptosis instigated by GSDME may elicit certain unfavorable responses in patients. Therefore, exploring the intricate relationship between other GSDM family proteins and tumors can open innovative avenues for tumor chemotherapy. Researchers should delve into refining combinations of pyroptosis-inducing agents with other treatment modalities, including immunotherapy, chemotherapy, or targeted therapies, aiming to generate synergistic effects that enhance overall treatment efficacy.

Future research in this field seeks to optimize the therapeutic potential of pyroptosis induction in cancer treatment while addressing challenges related to specificity, immune responses, and clinical applicability. As the understanding of pyroptosis deepens across various human disease domains, this programmed cell death pathway can profoundly impact cancer diagnostics and therapeutic interventions.

## Author contributions

JY: Writing – original draft, Writing – review & editing. JJ: Funding acquisition, Writing – review & editing.

## Glossary

**Table:**

GSDM	Gasdermin
GZM	Granzyme
ESCRT	Endosomal sorting complexes required for transport
NSCLC	Non-small cell lung cancer
ICIs	Immune checkpoint inhibitors
LPS	Lipopolysaccharide
PRRs	Pattern recognition receptors
PAMPs	Pathogen-associated molecular patterns
DAMPs	Damage-associated molecular patterns
HMGB1	High mobility group box-1
TLRs	Toll-like receptors
NLRs	Nucleotide-binding oligomerization domain-like receptors
AIM2	Absent in melanoma 2
ASC	Apoptosis-associated speck-like protein
NK cells	Natural killer cells
NLRP3	NOD-like receptor thermal protein domain associated protein 3
NETs	Neutrophil extracellular traps
PD-1/PD-L1	Programmed death-1/Programmed death ligand 1
GC	Gastric cancer
CRC	Colorectal cancer
eEF-2K	Elongation factor-2 kinase
ROS	Reactive oxidative species
JNK	c-jun N-terminal kinase
BRAF	B-Raf proto-oncogene
MEK	Mitogen-activated protein kinase
IFN-γ	Interferon γ
IECs	Intestinal mucosal epithelial cells
IBD	Inflammatory bowel disease
FAK	Focal adhesion kinase
ECM	Extracellular matrix
LC3B	Light chain 3B
CQ	Chloroquine
HER2	Human epidermal growth factor receptor 2
ccRCC	Clear cell renal cell carcinoma
DC	Dendritic cell
IRF2	Interferon regulatory factor 2
LUAD	Lung adenocarcinoma
LUSC	Lung squamous cell carcinoma
HCC	Hepatocellular carcinoma
SASP	Senescence-associated secretory phenotype
MLZE	Melanoma-derived leucine zipper extra-nuclear factor
TGF-β	Transforming growth factor β
TGF-βR2	Transforming growth factor-β receptor II
STAT3	Signal transducer and activator of transcription 3
NPs	Nanoparticles
CRS	Cytokine release syndrome
AP-1	Activator protein-1
CTLs	Cytotoxic T cells
MDSCs	Myeloid-derived suppressor cell
TAMs	Tumor-derived macrophages
DMF	Dimethyl fumarate
DSF	Disulfiram
MEG3	Maternally expressed gene 3
TNF-α	Tumor necrosis factor α
PLK1	Polo-like kinase 1
PDHK1	Pyruvate dehydrogenase kinase 1
